# Moving from idea to reality: The barriers and enablers to implementing Child and Family Hubs policy into practice in NSW, Australia

**DOI:** 10.1186/s12961-024-01164-0

**Published:** 2024-07-15

**Authors:** Anna Calik, Huei Ming Liu, Alicia Montgomery, Suzy Honisett, Kerri-Anne Van Munster, Tamara Morris, Valsamma Eapen, Sharon Goldfeld, Harriet Hiscock, John Eastwood, Susan Woolfenden

**Affiliations:** 1 Department of Community Paediatrics, Sydney Local Health District (SLHD), Sydney, NSW Australia; 2 Sydney Institute for Women, Children and Their Families, SLHD, Sydney, NSW Australia; 3 The George Institute for Global Health, University of New South Wales, Sydney, NSW Australia; 4 Sydney Medical School, The Faculty of Medicine and Health, University of Sydney, Sydney, NSW Australia; 5 Discipline of Psychiatry and Mental Health/Faculty of Medicine and Health, School of Clinical Medicine, University of New South Wales, Sydney, NSW Australia; 6 Discipline of Paediatrics and Child Health, University of New South Wales, Sydney, NSW Australia; 7 Department of Paediatrics, University of Melbourne, Victoria, Australia; 8 The Royal Children’s Hospital, Victoria, Australia; 9 Health Services and Economics, Centre for Community Child Health, Murdoch Children’s Research Institute, Victoria, Australia; 10 Centre of Research Excellence in Childhood Adversity and Mental Health, Centre for Community Child Health, Murdoch Children’s Research Institute, Victoria, Australia

**Keywords:** Childhood adversity, Health policy, Integrated health service, Integrated care, Scalability

## Abstract

**Background:**

Adverse childhood experiences can impact physical and mental health throughout the lifespan. To support families experiencing adversity and improve child health and developmental equity, an integrated, multi-sector response is required. Child and Family Hubs (Hubs) are a feasible and acceptable approach to providing such a response. In the Australian context, a number of federal and New South Wales (NSW) state policies support an integrated, multi-sector response using Hubs to support families experiencing adversity. This study examined NSW policy stakeholder and health service manager perspectives on the barriers and enablers to translating policy into practice in the implementation of Child and Family Hubs.

**Methods:**

Semi-structured interviews were conducted with 11 NSW government policy stakeholders and 13 community health service managers working in child and family policy and planning or child and family community-based services. Interviews were of 30–60 min duration and explored stakeholder knowledge, perspectives and experiences around childhood adversity, and barriers and enablers to operationalizing policies supporting Hubs. Analysis of barriers and facilitators to implementation of Hub models of care was undertaken using the Consolidated Framework for Implementation Research (CFIR).

**Results:**

Key barriers that emerged included short-term and inconsistent funding, lack of resourcing for a Hub co-ordinator, limited support for evaluation and insufficient time to plan for Hub implementation. Key enablers included flexibility and adaptability of Hub models to meet local needs, formal change management processes, strong governance structures and engagement among Hub practitioners. Key insights included the importance of targeted strategies to support sustained individual practice change and the need for organization-wide commitment to enable the successful adoption and maintenance of the Hub model of care.

**Conclusions:**

This study provides valuable insights and contributes evidence around what is needed to strengthen and support the operationalization and scalability of the Hub model of care. Key recommendations for Hub practitioners include the importance of formal change management processes and establishment of strong governance structures, while key recommendations for policymakers include the need for sustainable Hub funding and a standardized, evidence-based framework to support Hub implementation and evaluation.

**Supplementary Information:**

The online version contains supplementary material available at 10.1186/s12961-024-01164-0.

## Background

Adverse childhood experiences (ACEs) include exposure to adverse social determinants of health, such as community and peer dysfunction and socioeconomic disadvantage as well as experience of maltreatment and household dysfunction [1–3]. Exposure to ACEs has been shown to significantly impact physical and mental health throughout the lifespan, with multiple ACEs cumulatively impacting health risk [4,5]. Childhood adversities are common, with 52.8% of Australian children aged 10–11 years [6] and 75% of caregivers of children aged 0–8 years in New South Wales (NSW) reporting the experience of two or more adversities [7]. There is increasing evidence that positive childhood experiences, including stable and nurturing relationships and environments, and early interventions to identify and address childhood adversity can have a significant impact on health, wellbeing and productivity in adulthood despite exposure to ACEs [8,9].

Integrated care strategies seek to provide a multi-sector, co-ordinated response, including health, education and social support systems to improve health and wellbeing outcomes in children living with adversity [10,11]. This includes addressing barriers to service delivery, including fragmentation of services and complex referral pathways, through enhanced service collaboration. One approach to the delivery of integrated care to support families experiencing adversity is the Child and Family Hub model of care (Hubs) [12]. Hubs can provide improved access to a range of multi-disciplinary and intersectoral services, increasing opportunities for parental capacity-building and fostering of social connections [12]. Access to services can be facilitated through co-location and virtual service provision, with Hubs based in primary healthcare, Aboriginal Community Controlled Organizations, non-government organizations (NGOs), early childhood services and school settings [12]. While Hubs will vary depending on setting, emerging research suggests that core components to facilitate engagement include co-design with communities, family-centred care, parental capacity building, co-location of services, workforce development and local leadership [12]. Hubs have demonstrated benefits for child wellbeing, child development outcomes [13] and child mental health outcomes [14] and have been shown to be feasible [15] and acceptable and to facilitate improved access and engagement with services. [16]

Increasingly, Australian federal and state policy supports introduction of integrated care such as Hubs to support families experiencing adversity and promote child health, developmental and wellbeing outcomes [12]. Federal policies including the *National Children’s Mental Health and Wellbeing Strategy*, [17] *Safe and Supported: the National Framework for Protecting Australia’s Children* [18] and the *Draft Early Years Strategy* [19] support greater integration of services, multidisciplinary models of care and early intervention. At a state level, NSW has committed whole-of-government support to providing comprehensive and integrated services for children and families in the early years through policies including the *Brighter Beginnings initiative*. [20] This initiative aims to improve access to health information and integrated service delivery across health, education and family services, and includes early intervention programmes to support families experiencing adversity [20]. *Brighter Beginnings* is supported by the *NSW First 2000 Days Framework*, [21] highlighting strategies and interventions to promote health, wellbeing, capacity and resilience during early development, including additional services and supports to address adversity. Hubs are a key vehicle promoted in these strategies in terms of how such integrated approaches can be put into practice.

For effective translation in this favourable policy environment, it is essential to understand the key barriers and enablers to moving from policy to practice in Hub implementation. Emerging evidence from Hub evaluation identifies a number of key issues impacting operationalization, including funding, non-integrating information systems, leadership support and a shared vision among partner agencies [22]. A critical gap missing in Hub policy implementation is how to support the organizational and individual practitioner change required for Hubs to successfully run on the ground as a response to support families experiencing adversity.

The NSW health system is characterized by a devolved system of governance, with the NSW Ministry of Health developing health policy and setting standards of practice, and 15 local health districts responsible for policy implementation and health service delivery [23]. In this context, the insights of both NSW government policy stakeholders and community health service managers are essential to understanding the key barriers and opportunities to facilitate policy translation. The aim of this study was to examine NSW policy stakeholder and health service manager perspectives on the barriers and enablers to translating policy into practice in the implementation of Hubs to support families experiencing adversity.

## Methods

This study is part of the National Health and Medical Research Council and Beyond Blue funded Centre of Research Excellence in Childhood Adversity and Mental Health project, which aims to provide evidence to identify and support families experiencing adversity through the co-design, implementation and evaluation of two Child and Family Hubs in Victoria and NSW, Australia. [24]

### Participants

Participants were NSW government policy professionals (PPs) and community health service managers (CHSMs) working in child and family policy and planning or child and family community-based services. Participants were aged over 18 years and had sufficient English language proficiency to participate. Purposive sampling was used, with investigators J.E. and S.W., with experience working in government and Health sectors, nominating potential participants working in community health services and relevant government departments (NSW Ministry of Health, NSW Department of Education, NSW Department of Communities and Justice). Snowball sampling was used for subsequent recruitment, with each participant requested to refer further potential participants. Recruitment continued until data saturation was reached. Initial purposive sampling aimed to capture diverse perspectives from Health, Education and Social Services sectors and CHSMs working in government-funded and non-government organization-funded Hubs, however, as the focus of this paper is on Hubs providing health services, a high proportion of participants recruited were from the Health sector.

### Data collection

Potential participants were emailed a personalized invitation, specifying study objectives and a Participant Information Form. Due to COVID-19 pandemic workforce interruptions, interviews were undertaken between August 2021 and August 2023. A.C., A.M. and K.V. conducted semi-structured interviews using Zoom or Microsoft Teams video-conferencing platforms. Field notes were taken by hand. Interviews were transcribed verbatim and edited for clarity prior to analysis by A.C. and H.L. A.C. and H.L. both have a background in Public Health Medicine and HL a PhD in Health Systems Research and extensive qualitative research experience. A.M. and K.V. are paediatric clinicians. A.M. has extensive research experience in neurodevelopmental clinical trials and mixed-methods integrated care evaluation. All four researchers are female, with an interest in health equity. Interview duration was between 30 and 60 min. Non-participants were not present during interviews, and no repeat interviews were conducted. An interview guide was adapted from Honisett et al. [15] (see Additional file 1) and piloted by the study team. Interviews examined stakeholder knowledge and understanding of childhood adversity, and perspectives and experiences around barriers and enablers to operationalizing policies supporting the Hub model of care. Data collection processes are reported using the COREQ criteria for reporting qualitative research [25] (Additional file 2).

### Data analysis

Interview transcripts were imported into NVivo Release 1.7.1 for data management. Data were analysed using inductive and deductive framework analysis [26]. The draft coding framework for analysis was based on the Consolidated Framework for Implementation Research (CFIR) [27,28]. This is an implementation science framework that sets out a systematic approach to identifying potential barriers and facilitators to the successful implementation of an intervention [29]. The CFIR identifies five major domains within which barriers and enablers to implementation may occur: intervention characteristics, outer setting, inner setting, characteristics of individuals and implementation process [27,28]. Within each domain, the CFIR lists variables, termed “constructs”, that may impact implementation [27,28]. To explore the key barriers and facilitators to implementation of policies supporting Hub models of care, the results section is structured around the CFIR framework, with key themes presented under the five domains. A.C. developed a draft coding framework, based on the CFIR and a close coding of four initial interviews, through which emergent themes were inductively identified. H.L. independently coded the same four interviews, and A.C. and H.L. met to review and update the draft framework. As part of coding framework refinement, overlapping CFIR constructs and inductively identified themes were merged and adapted within the CFIR domain. A.C. applied the updated coding framework to the remaining transcripts.

### Ethics

Ethical approval was granted by The Royal Children’s Hospital Human Research Ethics Committee (HREC #62866) and Sydney Local Health District Human Research Ethics Committee (HREC #2020/ETH02883). Participants were provided a Participant Information Form, including information on how to withdraw from the study. Prior to each interview, participants provided informed verbal consent. To maintain confidentiality and anonymity, participants were provided the opportunity to decline use of exemplar quotes in publication(s) of study findings. Following each interview, transcripts were de-identified by the first author prior to coding to maintain anonymity. Participants were provided the opportunity to review and edit interview transcripts and comment on key findings. In response to participant concern regarding identifiability, limited demographic data are reported.

## Results

### Study participants

Thirty-two participants were invited, and eight declined to participate. Semi-structured interviews were conducted with 24 PPs and CHSMs, including 18 individual in-depth interviews and two focus groups. Eleven participants were NSW government PPs, and 13 were CHSMs. Participant demographics are presented in Table 1. Limited demographic information is provided due to participant concern regarding identifiability. Three participants worked in regional settings and 21 in metropolitan settings.

**Table 1:** Interview participants by sector and role

	NSW government policy professionals (PPs) (*n*)	Community health service managers (CHSMs) (*n*)
Health (*n*)	6	11
Education (*n*)	4	1
Social service (*n*)	1	1
Total (*n*)	11	13

### Dominant themes

**Fig. 1:**
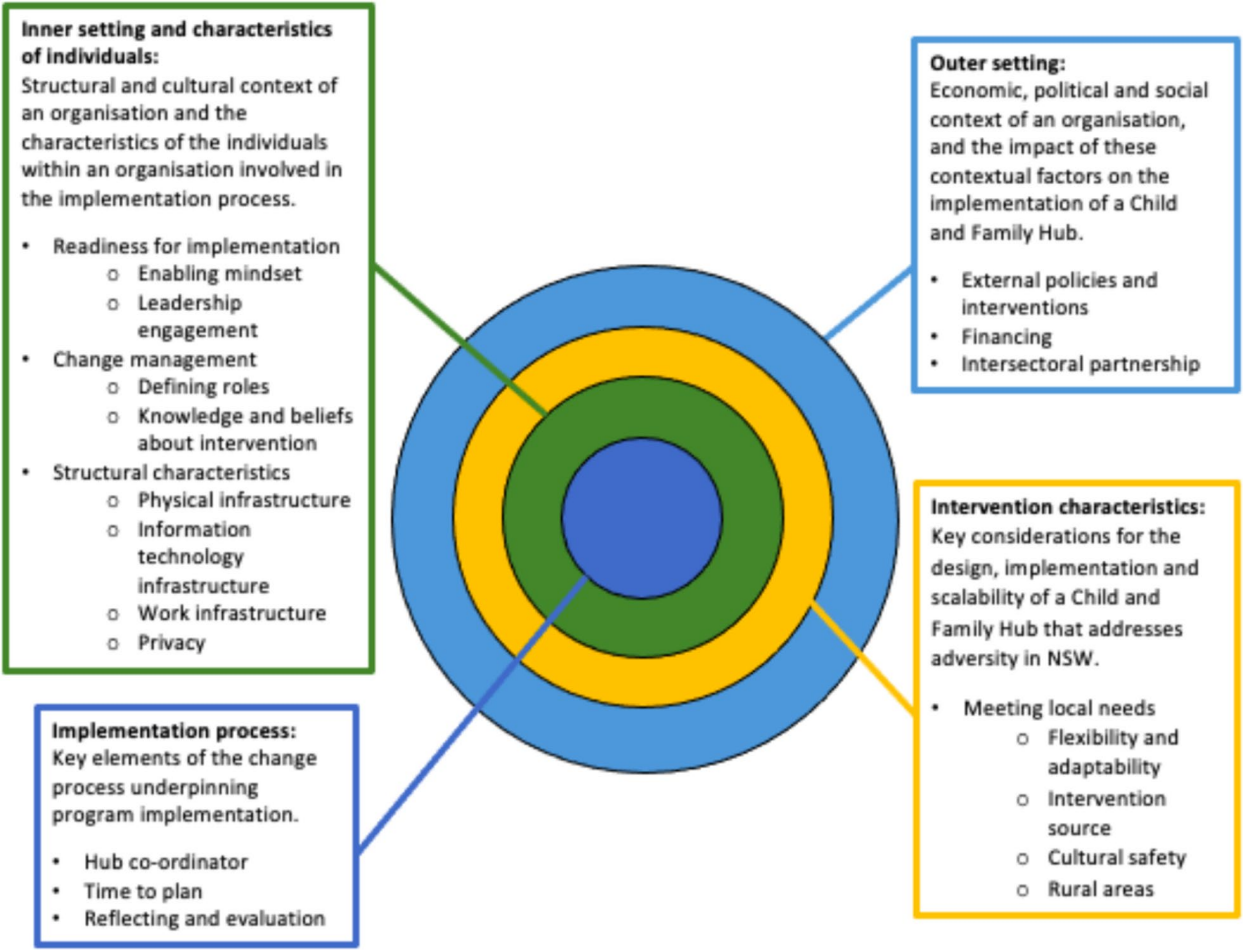
CFIR framework and key themes

Results are organized by CFIR domain. Figure 1 represents the domains as multi-level influences on implementation and provides a summary of each domain and emergent dominant themes and sub-themes.

### Intervention characteristics

The key theme in this domain was the necessity of meeting local needs. This referred to the need for flexibility in the Hub model of care to enable the design of initiatives responsive to local priorities. Sub-themes included flexibility and adaptability, co-design, cultural safety and suitability for rural areas.

#### Meeting local needs

- *Flexibility and adaptability*

Both PPs and CHSMs stated that, while Hubs could be implemented widely, this should not be as a “one-size-fits-all” model. Participants highlighted the necessity of flexibility and adaptability of the model to consider the diversity and circumstances of different communities and effectively respond to local family needs.

*So, talking about cultural safety and the flexibility of services and that they’re really understanding the needs of the communities that they’re delivering services to. So, it’s not just about well, we’ve got a service and you’ve got to fit in and around what we’re doing.* (PP9)

Participants also discussed the need for flexibility in referral criteria to provide holistic, family-centred care, including broader definitions of “family”, noting that “*the mother–child dyad doesn’t exist in a vacuum*” (CHSM1).

- *Intervention source: co-design*

Almost all participants spoke of the importance of undertaking co-design with the community to ensure that Hubs are designed to meet local priorities. It was noted that community input should not be limited to the design phase but that formal processes should be established to facilitate ongoing community feedback for continued service improvement.

*So then I would ask the community, and I think if you’re going to make these things work, I don’t think one model is ever going to fit everywhere. You have to co-design them for the local community.* (CHSM6)

- *Cultural safety*

The importance of designing and delivering services that are culturally safe for Aboriginal and Torres Strait Islander communities and Culturally and Linguistically Diverse communities was highlighted by all participants. It was emphasized that a community led design process is “…*particularly important for Aboriginal and Torres Strait Islander communities and multicultural communities because what we think looks right, it may not work for those communities.*” (CHSM10).

It was also emphasized that, for a service to be culturally safe for Aboriginal and Torres Strait Islander clients, it is essential to employ Aboriginal and Torres Strait Islander staff members, and that underpinning culturally safe service delivery is a culturally safe work environment:

*And that’s what you’ve got to look at. How do you make this a safe place for [Aboriginal and Torres Strait Islander] people to work in…it’s not just your clients, it’s the people who work for you as well. (CHSM12)*

- *Rural areas*

When considering the implementation of Hubs in rural areas, resourcing was highlighted as a significant issue. This included funding, but also availability and retention of appropriately trained staff.

*It’s very much dependent on what staff is available, not necessarily that this is the ideal team, it’s this is the available team…And so, you know, the policy implementation is so dependent on the workforce available at the time.* (PP4)

Virtual models of care to support implementation in rural areas were discussed, however, some limitations were noted, including that “*…not all, especially remote areas, have that kind of internet access, or families might not feel comfortable with those platforms.*” (CHSM13) Some participants described the use of supported telehealth models, for example, attending a local community health centre to connect to a virtual appointment, “…*and they* [clients] *feel quite comfortable with that.*” (CHSM13).

### Outer setting

Three key themes emerged under this domain: the role of external policies, financing and barriers to intersectoral partnership.

#### External policies

CHSMs emphasized the importance of policies such as the *First 2000 Days Framework* in expanding the parameters of what is considered the core business and responsibility of Health to include addressing childhood adversity. Both PPs and CHSMs noted that these policies provide services with the backing for practice changes some had already been making:

*So then, when the First 2000 Days framework came out, it was exactly what we were trying to say, and then people started talking about ACEs and childhood adversity, and maybe shifting the way they’re doing things.* (CHSM1)

However, CHSMs and some PPs discussed a number of limitations to these policies. These include that policies often contain broad “motherhood statements” without providing specific guidance around implementation, a lack of attached evaluation measures and an absence of resourcing to support their operationalization.

*They’re* [policies] *poorly communicated. They tend to be full of motherhood statements like we should all…provide timely care. But what does that mean?…so they’re not specific enough and there are no KPIs* [Key Performance Indicators] *attached to most of them.* (CHSM6)

#### Financing

CHSMs highlighted that “…*Hub services…are often funded on really, really soft money, and it's hard to do any long-term planning…*” (CHSM6). CHSMs noted that a successful Hub requires relationship building and ongoing relationship development, both with the local community and between Hub service providers, as well as capacity building among staff to support integrated ways of working. These processes can be challenging to undertake with short-term, insecure funding.

#### Intersectoral partnership

Both PPs and CHSMs discussed the importance of integration and intersectoral partnership and highlighted significant barriers to achieving this. This included that “…*we're not very good at working across our silos…health to mental health to social care to housing, everybody.*” (CHSM6).

It was noted that collaboration between multiple agencies from different sectors with different agendas, priorities, funding sources, performance indicators and organizational language “*requires quite a lot of work…and how you work through issues that come up is really quite tricky and it takes a lot of time and it's an ongoing process…*” (CHSM7). To address these challenges, participants highlighted “*…the need for that* [partnership] *to be core business, rather than a nice kind of add-on if you've got the time*” (PP9).

### Inner setting and characteristics of individuals

A number of key themes and sub-themes emerged in this combined domain focusing on organizational enablers to operationalizing policies and supporting individuals within an organization to adopt and maintain new models of care.

#### Readiness for implementation

An over-arching key theme in this domain focused on receptivity of individuals and organizational commitment to Hub implementation. In particular, the need for engagement from leadership and Hub practitioners to create a positive implementation climate were highlighted.

- *Leadership engagement*

CHSMs emphasized that “…*unless your CE* [Chief Executive] *is on board and you have high level sponsorship from your managers in Health and in other agencies, I think it’s really hard to do a Hub…*” (CHSM5). Leadership engagement was seen as a key factor in establishing organizational culture change to support integrated ways of working, and crucial in securing ongoing funding to support Hub sustainability.

- *Enabling mindset*

Both CHSMs and PPs highlighted the importance of buy in and a commitment to work together from all practitioners involved in the Hub.

*I guess another one* [enabler] *is enabling mindset, so all of the players, different departments, different NGO’s, all having the same idea that they want to collaborate, that they want to make this work, is a really big one.* (PP10)

Participants emphasized that implementing a Hub model of care *“…requires people to change the way they work*” (CHSM5), and while the importance of a shared vision and goal were discussed, readiness among Hub practitioners to establish new ways of working in an integrated way, and a willingness to work through the challenges associated with practice change were described as essential.

#### Change management

A second over-arching theme in this domain focused on the importance of formal change management processes to support organizational and individual change. Both CHSMs and PPs discussed the risk that following initial increased focus on changing practice due to a pilot, research or individual champion, with time, service providers may *revert* to previous practice:

*And while there was a focus on the pilot and the research, there was lots of effort being placed into integration, communication, collaboration…But once that kind of focus lifted, people, the practitioners tended to revert to old ways of operating.* (CHSM10)

Participants highlighted that good leadership is essential “*…to make sure that when the going gets tough, these services aren’t just going to revert back to what they used to do…leadership to support services…to keep them working in that integrated way*.” (CHSM1).

The need to create “*“framework, structures, scaffolds*” (PP10) to support long-term changes in practice was emphasized.

Two sub-themes emerged, including the need for formal opportunities for Hub practitioners to develop a shared understanding of each other’s roles and of the parameters of newly established Hub initiatives.

- *Defining roles*

CHSMs emphasized that co-location of services is not sufficient to achieve integration. It was noted that an important precursor to moving from siloed practice to working in an integrated way was to ensure Hub service providers had the opportunity to gain a greater understanding of each other’s roles and the services each can provide:

*And we had to do some work around, does everyone understand what each other actually does and do you know what the difference is between* [roles] *and…what skills do you actually bring…* (CHSM2)

- *Knowledge and beliefs about the intervention*

CHSMs spoke about the need to establish clear and agreed upon definitions of key terms such as “integration”, “care coordination”, “service navigation” and “case management” and developing a shared understanding of the parameters of the services being offered:

*… because those words mean different things to different clinicians, services, people...So, I think I would really want to focus on defining, on what that means for this Hub, and what are the limitations of it.* (CHSM1)

#### Structural characteristics

The final key theme in this domain focused on the importance of infrastructure supporting Hub implementation, including strong governance structures, the physical environment of the Hub and infrastructure to support information sharing.

- *Work infrastructure*

PPs and CHSMs emphasized the importance of formalizing the commitment to partnership and to implementing new models of care through clear governance, setting out key roles, responsibilities, organizational goals and the mechanisms through which these goals would be achieved. Participants noted that strong governance structures, “*with all your different partners involved, understanding their role in the Hub and being quite committed to it*” (CHSM5) are essential to ensure accountability and to sustain the changes to ways of working required to support integrated care.

- *Physical infrastructure*

Both PPs and CHSMs spoke about the need to create a welcoming, accessible environment that families will feel comfortable attending. It was also noted that the physical elements of the Hub, including configuration and design, can facilitate or be counterproductive to integrated and collaborative ways of working. The challenge of adapting pre-existing physical settings, compared with the opportunity to design and build a new physical setting for a Hub, was acknowledged.

*…there’s something that comes from the environment itself that makes a really big impact from what I’ve seen working in a Hub…really being thoughtful about environmental aspects…how it welcomes and invites people in and encourages the integrated approach…*(CHSM11)

- *Information technology infrastructure*

CHSMs identified use of non-linked information systems as a key barrier to integrated ways of working within a Hub. It was noted that even within an organization, service providers may be unable to view each other’s client notes due to different levels of access. A number of participants also discussed the under-utilization of existing systems, such as the electronic medical record, noting the opportunity to “…*improve its use, and really use it as a tool for integrated care…*” (CHSM1).

- *Privacy*

Participants highlighted the challenge of establishing organizational infrastructure that enables inter-agency knowledge sharing to support integrated care, while working within the boundaries of privacy legislation. The complexity of navigating this in intersectoral collaboration involving multiple government and non-government agencies was noted:

*So, we’d need to have a really strong understanding of the policies and processes around information sharing and privacy because you can’t do intersectoral work without that.* (CHSM1)

### Implementation process

Three key themes emerged in this domain: the importance of a Hub co-ordinator, time required to plan for Hub implementation and the importance and challenges of evaluation.

#### Hub co-ordinator

Both CHSMs and PPs identified the Hub co-ordinator role as integral to supporting the implementation and ongoing relationship management, integration and co-ordination of Hub services:

*The notion of funding, sometimes people call it the backbone, the sort of organising and co-ordinating role is pretty important because everybody’s stretched and somebody needs to do that extra work involving collaboration and pulling stuff together.* (PP2)

Some participants referred to this as a navigator role and highlighted a lack of agreement regarding responsibility for its funding given its intersectoral nature.

*…I think that independent navigator role is critical to crossing agencies, engaging other services…navigating with other services…Who funds that navigator role is the big question for me.* (CHSM8)

#### Time to plan

Participants highlighted the need to include time for relationship building and co-design with local communities in implementation timelines. CHSMs noted that typical timeframes for Hub implementation do not allow for a consultative and iterative planning process, and may result in an intervention that is not as effective as it could be:

*… you need government, or policy that allows people to do that planning and engagement work because…people might be given a sum of money and a target to implement something in two years or some kind of number…so, people start feeling the pressure to just get it going without really doing some of the planning and that community consultation.* (CHSM7)

Both PPs and CHSMs noted the importance of taking time to plan and consider location, ensuring a Hub is set up at “*an accessible site that a lot of people are going to be using anyway*” (PP2), rather than assuming a new Hub will draw people in and of itself.

#### Reflecting and evaluation

Both PPs and CHSMs discussed the importance of evaluation to demonstrate effectiveness in terms of patient outcomes, patient experience and cost-effectiveness. Participants stated that evidence of efficacy was integral in securing ongoing funding and support for an initiative, however, it was noted that “*It’s kind of hard to prove, cost-effectiveness in health is really difficult*” (PP3), particularly in the short term.

CHSMs also highlighted the importance of evaluation to contribute to the evidence base around Hub models of care, and support service quality improvement. The need to adequately resource robust evaluation was emphasized:

*…the recognition that good evaluation costs money and it needs to be paid for if we’re going to learn whether or not this is actually an effective way to support this target group.* (CHSM10)

CHSMs also noted the difficulty of undertaking programme evaluation while providing Hub services during time-limited appointments, and systems barriers due to the necessity of reporting different outcome measures via different portals to each funding body:

… *anything that’s funded through federal or state often want you to use their portals for data, but they all want you to do it differently and comment on different aspects…they take up a lot of time and they take you away from doing the work on the ground.* (CHSM11)

## Discussion

This study adds important evidence on the key barriers and enablers to operationalizing Hubs, especially in the policy ready environment of NSW. While previous studies have provided evidence of the feasibility of this model of care to identify and address adversity [15], this is the first study to utilize CFIR [27,28] to examine the challenges and enablers to its operationalization and scalability. In addition to contributing to the current evidence base on barriers to Hub implementation around funding, information-sharing infrastructure, Hub co-ordinator resourcing and evaluation support [15,22,30], we provide new insights into the importance of formal change management processes, engagement among Hub practitioners, enabling organizational structures and time to plan, strengthen intersectoral collaboration and support effective Hub implementation.

The key enablers participants highlighted around intervention characteristics were flexibility, adaptability and the importance of co-design to meet local needs. Co-design has previously been identified as a key component of Hubs [12,30], and while the co-design process has been shown to be feasible in this setting, adequate funding and time have been identified as challenges to this service design methodology [31]. Participants also identified the need for long-term and consistent funding to support Hub implementation, ongoing service delivery and evaluation, a finding consistent with key recommendations from a recent discussion paper examining the impact of integrated child and family centres in Australia [30]. Lack of organizational resources and insecure funding have been identified as key barriers to inter-organizational collaboration more broadly, including in youth care, mental health and primary care-based integrated care initiatives [32–34]. In international analyses of integrated mental health services, the operational unit of measure applied is the Basic Stable Input of Care, emphasizing the critical importance of stable resourcing to support stable staffing and thereby stable and consistent service delivery [35]. As highlighted by participants in the current study, lack of long-term, secure funding significantly undermines the opportunity for service growth and development, and system stability. A recent study examining the feasibility of the Hub model of care in the Community Health Services setting in Victoria, Australia, also highlighted the challenges of funding models such as activity-based funding, siloed funding and other inflexible funding approaches [15]. While these funding models were identified as a challenge by some participants in the current study, overwhelmingly, there was an emphasis on the implications of short-term funding on service provision, staff development and service planning. A number of participants discussed the precariousness of funding beyond the pilot phase, and this may account for the lesser focus in our findings on the longer-term concern of optimizing funding models to support integrated care.

What has not been described previously are the changes required to support individuals to work differently and organizations to adopt and maintain new models of care. Loss of intervention fidelity over time among health practitioners has been previously described [36,37], emphasizing the need for targeted strategies to support sustained practice change. Participants highlighted readiness for implementation within an organization as an essential precursor to establishing a Hub. The importance of leadership engagement and shared organizational goals for Hub sustainability has been discussed in a recent evaluation of a Hub initiative [22], but there has been limited focus in the Hub literature around the importance of change management processes, buy-in from all practitioners and commitment from service providers to undertake the work required to integrate service delivery. These factors, as well as addressing resistance to change, providing motivation for change and development of sustainability strategies have been identified as key to supporting successful organizational change in the business and management literature [38]. One example of how this can be put into practice comes from an Australian integrated care initiative focusing on vulnerable young people, vulnerable older people and people with chronic and complex conditions which demonstrated the benefit of multi-sectoral change management skills training in creating a common language and strengthening relationships among partner agencies [39]. These findings suggest the need for an increased focus on formal and informal organizational processes to support individuals to change their ways of working, enabling successful Hub implementation [40].

Our study also highlights the importance of enabling organizational structures to formalize commitment to partnership, support ongoing practice change and ensure accountability. The need for strong governance structures has been previously discussed in the Hub literature [22], and the broader literature on inter-organizational collaboration in integrated care [41]. The importance of clear, robust and sustained governance and oversight in multi-sectoral initiatives to ensure ongoing agency engagement, funding and programme sustainability has also been highlighted in a recent evaluation of a school-based integrated care initiative [42]. This suggests the potential utility of a standardized framework, to be implemented according to context, to support the development and evolution of all new and existing Hub initiatives through evidence-based guidance around governance structures and mechanisms to facilitate inter-organizational collaboration. Participants also highlighted the importance of physical infrastructure to facilitate more collaborative ways of working. While co-location is recognized as a key component of the Hub model of care [12], and virtual delivery of Hub services to increase access has been discussed [15], our findings suggest potential benefits to greater focus on the design of shared spaces and how these can facilitate engagement and relationship building between staff. The need for shared information technology infrastructure in Hubs has also been acknowledged [15,22,40], but a key barrier that emerged in our setting is the lack of clarity around information sharing processes that maintain privacy and confidentiality. Given the intersectoral nature of Hubs, involving collaboration between government and non-government agencies, development of clear processes for information exchange that meet current legislative and organizational policy requirements will be essential to support Hub operationalization. [43] This is an issue that has been previously identified in research examining an integrated care initiative supporting children in out-of-home care in NSW, Australia [44], highlighting this as a wider challenge to the provision of multi-disciplinary, multi-agency care for those experiencing complex and intersecting health and social needs.

Two key enablers identified that support Hub implementation and sustainability—dedicated resourcing for a Hub co-ordinator and evaluation—have been previously highlighted as integral to strengthening the impact of Hubs [30]. An additional key theme emerging from our work is the importance of time to plan for Hub implementation. Participants emphasized that flexible time frames are essential to support co-design, relationship building, Hub establishment and evaluation processes, while restricted timeframes can limit the efficacy and success of a Hub initiative. This is a key challenge with the issues described in terms of short-term funding of services and new programmes, and needs to be considered for any successful implementation of early years policies [30].

## Strengths and limitations

This study initially employed purposive sampling to select a diverse range of PPs and CHSMs. Snowball sampling was used for subsequent participant recruitment, with the potential for selection bias. To mitigate this, initial purposive sampling focused on the recruitment of a diverse range of participants from health, education and social services sectors, policy professionals from relevant government departments and CHSMs working in government-funded and non-government organization-funded Hubs, in rural and metropolitan areas. While this strategy was successful in recruiting participants from Health, Education and Social Services sectors and those working in regional and metropolitan areas, the majority of participants included in the study worked in the Health sector and were based in metropolitan settings. This may limit the generalizability of these findings, particularly in regards to Hubs in rural and regional areas, and Hubs based outside of Health settings. However, there was a general consistency in the key barriers and facilitators to Hub implementation identified by participants across sectors and settings. Additionally, it is noted that 8 of the 32 participants invited to the study declined to participate. No reasons were provided. Our study findings may have been impacted if a disproportionate number of participants who elected to participate in the study were those who worked in Hubs experiencing operational challenges, whereas those who declined to participate worked in Hubs with fewer challenges, resulting in an under-identification of facilitators to Hub implementation. Underlying causes for challenges to implementation may also have been misattributed by participants. Additionally, the predominance of participants from the Health sector may have led to an over-emphasis of issues affecting Hubs based in Health settings. However, a number of the barriers and facilitators identified in the current study are consistent with those previously identified in the Hub literature and the wider literature on inter-organizational collaboration in integrated care, lending support to our findings. It is recommended that future research further explore the perspectives of PPs and CHSMs in Education, Social Service and Legal sectors and regional areas. A key strength of our study is use of the CFIR framework to provide a focused examination of the key challenges and facilitators to Hub implementation. While this study captures valuable insights and perspectives of PPs and CHSMs in NSW, further implementation of science-focused research around barriers and enablers to Hub operationalization in other jurisdictions is recommended to facilitate a wider approach to scaling of this model of care.

## Implications for policy and practice

This study adds to the existing knowledge gap in Hub policy implementation research around how to support the organizational and individual practitioner change required for successful Hub implementation and sustainability. It is novel in bringing together the perspectives of PPs and CHSMs in the policy ready environment of NSW, and utilizing the CFIR [26,27] to identify key barriers and enablers to service- and system-level change and highlight opportunities to optimize the planning, implementation and evolution of new and existing Hubs.

Based on our findings, key recommendations for Hub practitioners to support initiative sustainability include: the importance of formal change management process, such as multi-sector change management skills training, to engage Hub practitioners and strengthen relationships; establishment of strong governance structures to formalize commitment to partnership, support ongoing practice change and ensure accountability; development of clear processes for information exchange that maintain privacy and confidentiality; consideration of physical spaces in the design of a Hub and how these can facilitate collaboration; and taking the time to plan, including undertaking co-design processes to ensure initiatives meet local needs.

Key recommendations for policymakers include the following: the need for stable, long-term funding to support sustainable Hub initiatives; support for research to develop the evidence around best practice for the Hub model of care; and the need for a standardized framework to provide evidence-based guidance around Hub implementation and evaluation. The National Child and Family Hubs Network [45] (the Network) is a national, multidisciplinary group with a focus on strengthening and supporting Hubs across Australia. The Network aims to advance research around best practice in Hub design, implementation and evaluation; ensure availability of evidence and resources to build collective capacity; and develop an implementation and evaluation framework for Hubs.^45^ The current study highlights the importance of resourcing such work to ensure that new and evolving Hubs are effectively supported to successfully deliver sustainable programmes to support families and children experiencing adversity.

## Conclusions

Understanding the key barriers and enablers to policy translation of Hubs to support families experiencing adversity is essential to changing the way services are delivered to better support families and children. This could reduce inequity, improve health and wellbeing across the lifespan, and provide more cost-effective early intervention. Our study highlights the importance of long-term sustainable funding, standardized frameworks to support implementation and evaluation, targeted strategies to support sustained individual Hub practitioner and organizational change, and strong governance structures to formalize commitment to partnership and ensure accountability as key to strengthening and effectively supporting the operationalization and scalability of the Child and Family Hub model of care.

### Supplementary Information

Additional file 1.

Additional file 2.

Additional file 3.

Additional file 4.

## Data Availability

The datasets generated and/or analysed during the current study are not publicly available due to privacy provisions but are available from the corresponding author on approval from the authorizing ethics committee.

## Abbreviations

ACEs: Adverse childhood experiences
CFIR: Consolidated Framework for Implementation Research
CHSM: Community health service manager
NGO: Non-government organization
NSW: New South Wales
PP: Policy professional
