# Supplementary material for: Moving from idea to reality: The barriers and enablers to implementing Child and Family Hubs policy into practice in NSW, Australia
Source: Health Res Policy Syst. 2024 Jul 15;22:83. doi: 10.1186/s12961-024-01164-0 (PMC11247851; doi:10.1186/s12961-024-01164-0)
Supplement: Supplementary file 1 — Additional file 1. [file 12961_2024_1164_MOESM1_ESM.docx]

**Additional file 1: Interview guide**

| **Concept** | **Questions** |
| --- | --- |
| 1. Introduction | 1.1 Could you tell me a bit about your role and the key focus areas of your work? |
| 2. Knowledge and understanding of childhood adversity and mental health | 2.1 What key pieces of work are you involved with that relate to childhood adversity or child mental health or both? |
| 3. Current policy and service receptivity for extending the hub pilot into broader state-wide rollout | 3.1 Our team have identified some policies across NSW that relate to childhood adversity and/or mental health and/or integrated care.  [interviewee shown policy list slide] (Additional file 3)  Do you think that these policies are the primary ones that would support integrated services, such as child and family hub models of care, across the state? |
|  | 3.2 What other policies are relevant to the child and family hub model in this state? |
| 4. Opportunities and barriers for operationalising child and family hubs within community health services | 4.1 Tell me your thoughts on available approaches to operationalising these policies that support child and family hub models of care? |
|  | 4.2 What are some of the opportunities or enablers for this? |
|  | 4.3 What are some of the barriers for this? |
| 5. Feedback on the proposed child and family hub model | [Interviewee shown a model of proposed key components of a child and family hub including:   - Intersectoral linkages - Early detection and assessment - Case discussions - Service navigation/social prescribing - Workforce development - Referral pathways - Evidence based interventions]   5.1 Could this model work for Community Health Services state-wide? |
|  | 5.2 Would this model be appropriate to be implemented in rural areas? |
|  | 5.3 What components of the model could happen remotely? |
| 6. Policy levers to support broader scaling up of the child and family hub model | 6.1 What would be required from government to support the scale up of child and family hubs across NSW? |
