# Supplementary figures and images for: Moving from idea to reality: The barriers and enablers to implementing Child and Family Hubs policy into practice in NSW, Australia
Source: Health Res Policy Syst. 2024 Jul 15;22:83. doi: 10.1186/s12961-024-01164-0 (PMC11247851; doi:10.1186/s12961-024-01164-0)

**Additional file 2: COREQ Consolidated Criteria for Reporting Qualitative Research Checklist**
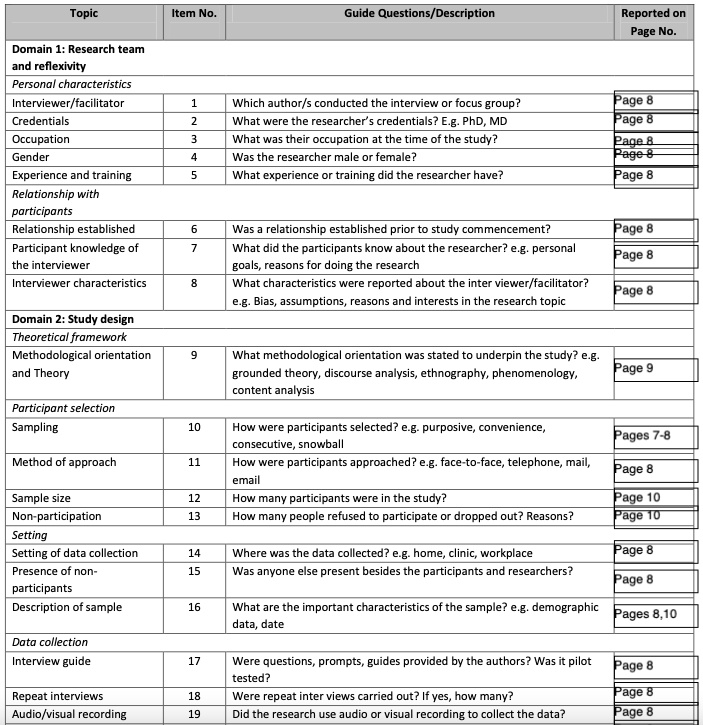

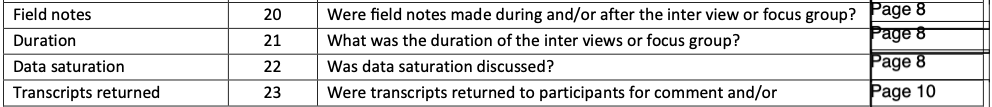


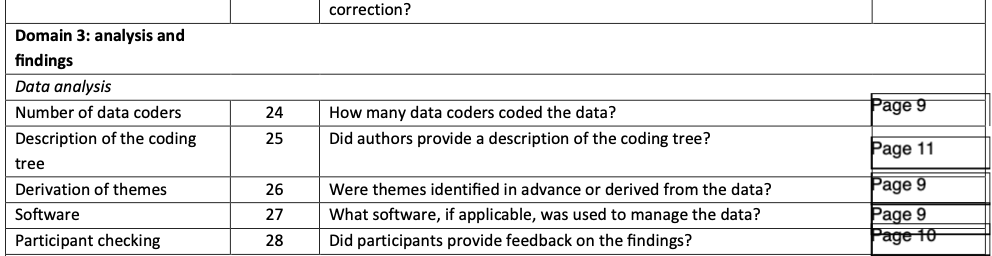


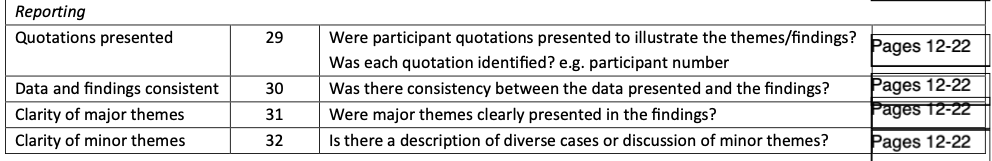

Supplement: Supplementary file 2 — Additional file 2. [file 12961_2024_1164_MOESM2_ESM.docx]
