# Supplementary material for: Moving from idea to reality: The barriers and enablers to implementing Child and Family Hubs policy into practice in NSW, Australia
Source: Health Res Policy Syst. 2024 Jul 15;22:83. doi: 10.1186/s12961-024-01164-0 (PMC11247851; doi:10.1186/s12961-024-01164-0)
Supplement: Supplementary file 3 — Additional file 3. [file 12961_2024_1164_MOESM3_ESM.docx]

**Additional file 3: Policies that could support Child and Family Hubs in NSW**

| **National Policies** | - Safe and Supported: The National Framework for Protecting Australia’s Children 2021-2031 - National Disability Insurance Scheme - Supporting Families, Communities and Organisations to Keep Children Safe: Fourth Action Plan - Families and Children Activity - Families and Communities Program - Mental Health Report, Productivity Commission - National Action Plan for the Health of Children and Young People 2020-2030 - National Children’s Mental Health and Wellbeing Strategy - Australia’s Disability Strategy 2021-2031 – Early Childhood Targeted Action Plan - Australia’s Disability Strategy 2021-2031 – Early Childhood Targeted Action Plan - National Strategic Framework for Aboriginal and Torres Strait Islander Peoples’ Mental Health and Social and Emotional Wellbeing - National Aboriginal and Torres Strait Islander Health Plan - National Agreement on Closing the Gap - Royal Australasian College of Physicians Inequities in Child Health Position Statement - United Nations Convention on the Rights of the Child |
| --- | --- |
| **National Legislation** | - Disability Discrimination Act - Disability Standards for Education |
| **NSW Policies** | - Supporting Families Early Package- Maternal & Child Health Primary Health Care Policy - Building Strong Foundations - First 2000 Days Framework - Integrated Prevention and Response to Violence, Abuse & Neglect Framework - Child Wellbeing and Child Protection Policies and Procedures for NSW Health - The Henry Review and Implementation Plan |
| **NSW Legislation** | - Children and Young Persons (Care and Protection) Act |
| **NSW Programs** | - SAFE Start - Universal Health Home Visiting - Sustained Health Home Visiting - Statewide Eyesight Preschooler Screening (StEPS) Program - State Wide Infant Screening Hearing (SWISH) Program - Aboriginal Ear Health Program - Child Protection Counselling Service - Out of Home Care Health Pathway Program - Brighter Beginnings |
