# Supplementary material for: Moving from idea to reality: The barriers and enablers to implementing Child and Family Hubs policy into practice in NSW, Australia
Source: Health Res Policy Syst. 2024 Jul 15;22:83. doi: 10.1186/s12961-024-01164-0 (PMC11247851; doi:10.1186/s12961-024-01164-0)
Supplement: Supplementary file 4 — Additional file 4. [file 12961_2024_1164_MOESM4_ESM.docx]

**Additional file 4: Coding framework**

| **Domain** | **Theme/Sub-theme** | **Definition** | **Notes** |
| --- | --- | --- | --- |
| **Intervention characteristics** Characteristics of the intervention being implemented into a particular setting. | Meeting local needs (inductive theme) | Interventions need to be flexible and adaptable to meet local needs | Major theme |
|  | Patient needs and resources | The extent to which patient needs, as well as barriers and facilitators to those needs, are accurately known and prioritised by the organisation, including organisational cultural competence and provision of culturally safe services. | Overlap with meeting local needs *In outer setting in CFIR model |
|  | Sub-theme: Intervention flexibility and adaptability | Degree to which an intervention can be adapted, tailored, refined, or reinvented to meet the needs of local communities. | Sub-theme |
|  | Sub-theme: Referral criteria (inductive theme) | Criteria for referral to and eligibility to access intervention | Overlap with "intervention flexibility and adaptability" |
|  | Sub-theme: Intervention source: co-design | Perception of key stakeholders about whether the intervention is externally or internally developed. *includes co-design with local community | Sub-theme |
|  | Sub-theme: Cultural safety (inductive theme) | Ensuring provision of culturally safe and culturally appropriate services. | Sub-theme |
|  | Sub-theme: Rural areas (inductive theme) | Implementation issues/barriers/challenges in regional, rural and remote settings | Sub-theme |
| **Outer setting** Economic, political and social context within which an organisation resides. | External policies | Broad constructs that encompass external strategies to spread interventions including policy, regulations, external mandates, recommendations, guidelines, public or benchmark reporting. | Major theme |
|  | Financing | Funding from external entities to support implementation of intervention. Also consider sustainability of funding model. | Major theme |
|  | Sub-theme: Hard sell (inductive theme) | Difficult to get long-term funding and commitment for an initiative that will only see outcomes in the long term | Overlap with financing |
|  | Intersectoral partnerships | The degree to which an organisation is networked with other external organisations. | Major theme  *Cosmopolitanism in CFIR model |
|  | Sub-theme: Collaboration across health services (inductive theme) | Linkage, communication, collaboration with other health services/providers (outside of hub but within health). | Overlap with "intersectoral partnerships" |
|  | Sub-theme: Service integration (inductive theme) | Communication and connectivity between hub service providers to enable provision of seamless, effective and efficient care | Overlap with "intersectoral partnerships" |
| **Characteristics of individuals** Individuals involved with the intervention and/or implementation process. | Change management (inductive theme) | Formal process to support implementation and organisational and individual change. | Major theme |
|  | Sub-theme: Defining roles (inductive theme) | Clear and shared understanding of own and others' roles and responsibilities | Sub-theme |
|  | Sub-theme: Knowledge and beliefs about the intervention | Individuals' attitudes toward and value placed on the intervention as well as familiarity with facts, truths, and principles related to the intervention. | Sub-theme |
|  | Sub-theme: Reverting to previous practice (inductive theme) | Tendency to revert to previous practice/roles/ways of doing things | Sub-theme |
| **Inner setting** Features of structural, political and cultural contexts through which the implementation process will proceed. | Implementation climate | The absorptive capacity for change, shared receptivity of involved individuals to an intervention, and the extent to which use of that intervention will be rewarded, supported and expected within their organisation. | Overlap with "Readiness for implementation" |
|  | Readiness for implementation | Tangible and immediate indicators of organisational commitment to its decision to implement an intervention, consisting of 3 subconstructs (leadership engagement, available resources, access to information and knowledge) | Major theme |
|  | Sub-theme: Enabling mindset (inductive theme) | Buy in from all involved actors | Sub-theme |
|  | Sub-theme: Leadership engagement | Commitment, involvement and accountability of leaders and managers with the implementation. | Sub-theme |
|  | Sub-theme: Available resources | The level of resources dedicated for implementation and ongoing operations including money, training, education, workforce capacity, physical space and time. | Overlap with "financing" in outer setting |
|  | Sub-theme:  Access to information and knowledge | Ease of access to digestible information and knowledge about the intervention and how to incorporate it into work tasks. | Overlap with "knowledge and beliefs about the intervention" in characteristics of individuals |
|  | Structural characteristics | Infrastructure components that support functional performance, consisting of 3 subconstructs (physical, information technology and work infrastructure). | Major theme |
|  | Sub-theme: Physical infrastructure | Layout and configuration of space and other tangible material features. | Sub-theme |
|  | Sub-theme: Information technology infrastructure | Technological systems for communication, electronic documentation, data storage, management, reporting and analysis. | Sub-theme |
|  | Sub-theme: Work infrastructure | Organisation of tasks and responsibilities within and between individuals and teams, including clear governance structures. | Sub-theme |
|  | Sub-theme: Privacy (inductive theme) | Clear understanding of, and working within, policies around privacy and confidentiality in regards to sharing of patient information | Sub-theme |
| **Process** Implementation process. Successful implementation usually requires an active change process aimed to achieve individual and organisational level use of the intervention as designed. | Champion | Change agent/leader to champion and promote paradigmatic change to facilitate changed thinking/changing policy environment. | Overlap with "internal implementation leaders" |
|  | Internal implementation leaders - "Hub co-ordinator" | Formally appointed co-ordinator, project manager, team leader. | Major theme |
|  | Planning - "Time to plan" | The degree to which a scheme or method of behaviour and tasks for implementing an intervention are developed in advance and the quality of those schemes or methods. | Major theme |
|  | Reflecting and evaluation | Quantitative and qualitative feedback about the progress and quality of implementation accompanied with regular personal and team debriefing about progress and experience. | Major theme |
